# Importance of *TP53 codon 72 and intron 3 duplication 16bp *polymorphisms in prediction of susceptibility on breast cancer

**DOI:** 10.1186/1471-2407-8-32

**Published:** 2008-01-29

**Authors:** Sandra Costa, Daniela Pinto, Deolinda Pereira, Helena Rodrigues, Jorge Cameselle-Teijeiro, Rui Medeiros, Fernando Schmitt

**Affiliations:** 1 Life and Health Sciences Research Institute, School of Health Science, University of Minho, Braga, 4710-057, Portugal; 2 Molecular Oncology Unit, Portuguese Institute of Oncology – Porto, Porto, 4200-072, Portugal; 3 Hospital Xeral-Cíes, Vigo, Spain; 4 ICBAS, Abel Salazar Institute for the Biomedical Sciences, Porto, 4099-003, Portugal; 5 Medical Faculty of Porto University and IPATIMUP, Institute of Molecular Pathology and Immunology of the University of Porto, Porto, 4200-465, Portugal

## Abstract

**Background:**

*TP53 *is one of major tumour suppressor genes being essential in preservation of genome integrity. Two very common polymorphisms have been demonstrated to contribute to cancer susceptibility and tumour behaviour. The purpose of this study was to evaluate the role of *Arg72Pro *and *PIN3 Ins16bp *polymorphisms in *TP53 *gene as genetic susceptibility and predictive markers to breast cancer.

**Methods:**

We analysed DNA samples from 264 breast cancer patients and 440 controls, for *TP53 Arg72Pro *and *PIN3 Ins16bp *polymorphisms using PCR-RFLP.

**Results:**

We observed that women with *A2A2 *genotype have increased risk for developing breast cancer, either in women with or without familial history (FH) of the disease (OR = 4.40, 95% CI 1.60–12.0; p = 0.004; OR = 3.88, 95% CI 1.18–12.8; p = 0.026, respectively). In haplotype analysis, statistically significant differences were found between *TP53 Arg-A2 *haplotype frequencies and familial breast cancer cases and the respective control group (OR = 2.10, 95% CI 1.08–4.06; p = 0.028). Furthermore, both *TP53 *polymorphisms are associated with higher incidence of lymph node metastases.

**Conclusion:**

Our findings suggest *TP53 PIN3 Ins16bp *polymorphism as a real risk modifier in breast cancer disease, either in sporadic and familial breast cancer. Furthermore, both TP53 polymorphisms are associated with higher incidence of lymph node metastases.

## Background

Breast cancer have been associated with well-established risk factors, such as high estrogen exposure, environmental factors (e. g. diet and ionizing radiation) and family history [1,2]. Family history of breast cancer is a particularly important high risk factor for this disease. Two genes were identified as the major susceptibility genes in high risk families, namely *BRCA1 *and *BRCA2*. However, these genes account for only a minority of the overall family risk of breast cancer [3]. Furthermore, approximately only 10% of all breast cancer cases exhibit a familial pattern of incidence [4,5]. In this way, the remaining familial and sporadic risk may be due to common low to moderate penetrance genetic variants, which are also referred as genetic polymorphisms. One strong candidate for genetic susceptibility factor to familial and/or sporadic breast cancer is the *TP53 *gene. This gene is frequently somatically mutated in breast cancer [6,7] and *TP53 *germline mutations are associated with increased risk for developing diverse malignancies, including 25–30% of hereditary breast cancer cases associated with Li-Fraumeni syndrome [8]. Furthermore, based on its pivotal role in DNA damage repair and its physical and functional interactions with BRCA1 and BRCA2 proteins [9,10], *TP53 *seems to be a strong candidate breast cancer predisposition.

The *TP53 *tumour suppressor gene, also designated the guardian of the genome, is essential in preservation of genome integrity. From the numerous biological functions of p53 protein, inhibition of cell cycle progression, DNA repair and apoptosis are the major cellular pathways where it is involved [6].

*TP53 *gene mutations are widely detected in breast cancer, being correlated with specific clinical phenotypes [11,12].

Predisposition to several human cancers has been associated with genetic polymorphisms, which may represent an important contribution to cancer susceptibility and tumour behaviour [13-16]. Several polymorphisms have been identified within *TP53 *gene, both in non-coding and coding regions [17]. One of the most well studied *TP53 *gene polymorphism is *Arg72Pro*, located in codon 72 on exon 4, leading to arginine-proline substitution, which in its turn results in a structural alteration of the protein [18]. Another common polymorphism is 16 base pair (bp) duplication in intron 3 of the *TP53 *gene (*PIN3 Ins16bp*).

In this case-control study, we hypothesize that the two common polymorphisms of *TP53 *gene play a role either apoptosis, cell cycle control efficiency, as well as DNA repair capacity, which ultimately may contribute to an increase of breast cancer susceptibility within familial and/or sporadic cases, as well as represent an additional tool for prognosis prediction.

## Methods

### Study Population

We analysed a total of 264 DNA breast cancer cases: 73 unrelated familial breast cancer cases were selected from the Oncology and Surgical Departments from S. João Hospital at Porto and Vigo Hospital, and 191 unrelated sporadic breast cancer cases were recruited from IPO-Porto (Oncology Portuguese Institute), during 1998–2003, from patients that were receiving treatment. All cases were histological confirmed at the Department of Pathology. Clinico-pathological parameters were obtained when possible from hospital clinical records. Familial case group presented a mean age of 42.07 years, with an age range of 24–77 years. The high-risk familial breast cancer group, also designated by us as family history (FH) breast cancer cases, included women with the follow features, based on the Breast Cancer Linkage Consortium criteria [19]: early onset (≤40 years) and/or bilaterality; or more than three cases of breast cancer in the family; or more than one case of ovarian cancer in the family; or more than two first-degree relatives involved; or male breast cancer. These high-risk breast cancer cases are *BRCA1/BRCA2 *mutations non-carriers [20]. Sporadic cases group (with no presence of FH) presented a mean age of 53.41 years, with an age range of 41–88 years. Control women were randomly selected from blood banks in the same region during the same time period as the cases were collected. The selection criteria include no prior history of cancer, and controls were frequency matched to the cases by age (± 5 years). A total of 440 healthy women presenting a median age of 42.29 years and an age range of 21–85 years, were used as control group of familial breast cancer cases. From the above control group 216 healthy women were selected according to the age of diagnosis of the sporadic breast cancer patients group (higher than 40 years), with a median age of 53.05 and an age range of 41–85, being used as control group of sporadic breast cancer cases. All participants provided informed consent. Ethical approval was obtained by the specific institutions ethical committees.

### Laboratory Methods

Genomic DNA was isolated from lymphocytes of peripherical blood using Puregene^® ^DNA Purification Kit (Gentra Systems, Minneapolis, USA). All the polymorphisms were assessed by PCR-RFLP technique. *TP53 Arg72Pro *polymorphism (rs1042522) was detected by amplifying genomic DNA with the forward primer 5'-GAA GAC CCA GGT CCA GAT GA-3' and the reverse primer 5'-CTG CCC TGG TAG GTT TTC TG-3'. The PCR amplification parameters were 32 cycles each of 30 sec at 94°C, 30 sec at 54°C, and 30 sec at 72°C. The 152 bp PCR product was digested with *Bsh1236I *(Fermentas, Ontario, Canada) at 37°C overnight. Digested products were separated by electrophoresis in a 3% agarose gel (Seakem^® ^LE Agarose, Rockland, USA) and visualized by ethidium bromide staining. Wild type alleles resulted in 50 and 102 bp fragments and the variant alleles resulted in 152 bp fragment following restriction enzyme digestion. *TP53 PIN3 Ins16bp *polymorphism (rs17878362) was detected by amplifying genomic DNA with the forward primer 5'-CTG AAA ACA ACG TTC TGG TA-3' and the reverse primer 5'-AAG GGG GAC TGT AGA TGG GTG-3'. The PCR amplification parameters were 32 cycles each of 30 sec at 94°C, 30 sec at 60°C, and 30 sec at 72°C. The PCR product was separated by electrophoresis in a 4% agarose gel (Seakem^® ^LE Agarose, Rockland, USA) and visualized by ethidium bromide staining. Wild type alleles, designated *A1 *allele (no duplication) resulted in 119 bp fragment and the variant alleles, designated *A2 *allele (with 16 bp duplication) resulted in 135 bp fragment.

To ensure quality control of all genotyping results, 10% of the samples was randomly selected and sequenced using an ABI automated sequencer. We obtained a 98% of concordance between the genotyping results acquire by the two methods.

### Statistical Analysis

Analysis of data was performed using the computer software SPSS version 14.0 (SPSS Inc., Chicago, USA). Chi-square (χ2 test) analysis was used to compare categorical variables. Whenever necessary, the Fisher test was used when number of samples was equal or inferior to 5. A 5% level of significance was used in the analysis. The Odds Ratio (OR) and its 95% confidence interval (CI) were calculated to measure the association between *TP53 *polymorphic genotypes and breast cancer risk. Logistic regression analysis was used to calculate the adjusted OR and 95% CI for the influence of *TP53 *genotypes in the risk of breast cancer, adjusted for age and/or FH. Whenever appropriate, the observed number of each genotype in control groups were compared with that expected for a population in the Hardy-Weinberg Equilibrium by using a goodness of fit χ2 test. The frequencies of expected haplotypes were estimated by using the statistical methodologies implemented by HPlus software [21], which infers haplotypes based on expectation-maximization with a modified progressive ligation computational algorithm. The nonparametric test, Kruskal-Wallis test, was used to compare mean age of diagnosis between the different genotype polymorphisms.

## Results

The distribution of the genotype frequencies in *PIN3 Ins16bp *polymorphisms among control group (p = 0.478) and in *Arg72Pro *and *PIN3 Ins16bp *among control subgroup (p = 0.082 and p = 0.294) is in agreement with those expected under Hardy-Weinberg equilibrium, excepted for *Arg72Pro *in the overall control group (p = 0.013).

Concerning *TP53 Arg72Pro *polymorphism in the familial breast cancer cases, frequencies of *Arg72Arg*, *Arg72Pro *and *Pro72Pro *were 53.4%, 34.2% and 12.3%, respectively. In sporadic breast cancer, 56.0%, 34.9% and 9.1% were homozygous for *72Arg *allele, heterozygous and homozygous for *72Pro *allele, respectively. No statistically significant associations were found between the *TP53 Arg72Pro *polymorphism and risk of familial and sporadic breast cancer risk (Table 1).

**Table 1:** *TP53 Arg72Pro *and *PIN3 Ins16bp *genotypic and allelic frequencies. Association with familial and sporadic breast cancer risk.

TP53 Polymorphism	Genotype	Positive FH Cases	Controls	OR* (95% CI)	Sporadic Cases	Controls	OR* (95% CI)
*Arg72Pro*							
	*Arg/Arg*	39 (53.4)	256 (59.0)	Reference	98 (56.0)	124 (58.5)	Reference
	*Arg/Pro*	25 (34.2)	142 (32.7)	1.19 (0.68–2.08)	61 (34.9)	70 (33.0)	1.26 (0.79–2.02)
	*Pro/Pro*	9 (12.3)	36 (8.3)	1.58 (0.68–3.67)	16 (9.1)	18 (8.5)	1.35 (0.63–2.88)
Alleles							
	*Arg*	103 (70.5)	654 (75.3)	Reference	257 (73.4)	318 (75.0)	Reference
	*Pro*	43 (29.5)	214 (24.7)	1.28 (0.85–1.91)	93 (26.6)	106 (25.0)	1.09 (0.78–1.52)

*PIN3 Ins16bp*							
	*A1A1*	46 (65.7)	299 (68.0)	Reference	122 (63.9)	147 (68.1)	Reference
	*A1A2*	15 (21.4)	130 (29.5)	0.80 (0.43–1.49)	56 (29.3)	65 (30.1)	1.07 (0.67–1.70)
	*A2A2*	9 (12.9)	11 (2.5)	4.40 (1.60–12.0)	13 (6.8)	4 (1.9)	3.88 (1.18–12.8)
Alleles							
	*A1*	107 (76.4)	728 (82.7)	Reference	300 (78.5)	359 (83.1)	Reference
	*A2*	33 (23.6)	152 (17.3)	1.48 (0.94–2.31)	82 (21.5)	73 (16.9)	1.34 (0.93–1.94)

* OR adjusted for age (logistic regression analysis)

FH – family history; OR – odds ratio; CI – confidence interval

Frequencies of *TP53 PIN3 Ins16bp *polymorphism genotypes were 65.7% to *A1A1*, 21.4% to *A1A2 *and 12.9% to *A2A2*, in familial breast cancer cases. Regarding sporadic breast cancer group, we observed 63.9%, 29.3% and 6.8% frequencies for homozygous for *A1 *allele, heterozygous and homozygous for *A2 *allele, respectively. We observed that *A2A2 *genotype carriers with positive FH were at a 4.40-fold (95% CI = 1.60–12.0; p = 0.004) increased risk of breast cancer compared with the respective control group. Moreover, statistically significant differences were observed in *A2A2 *genotype frequencies comparing sporadic breast cancer cases and respective control group (p = 0.026). Our results showed that carriers of *A2A2 *genotype with no FH present an increased risk of breast cancer (OR = 3.88, 95% CI 1.18–12.8).

We investigated haplotype effects of the two polymorphisms studied in breast cancer risk (Table 2). Compared the common *TP53 Arg-A1 *haplotype with the other expected haplotypes; we only observed statistically significant differences regarding *TP53 Arg-A2 *haplotype between the familial breast cancer cases and respective control group (p = 0.028). Carriers of *TP53 Arg-A2 *haplotype and presence of FH of breast cancer presented an increased risk of develop breast cancer (OR= 2.10; 95% CI 1.08–4.06).

**Table 2:** Expected haplotype frequencies between *Arg72Pro *and *PIN3 Ins16bp *polymorphisms. Association with familial and sporadic breast cancer risk.

Haplotypes	Positive FH Cases	Controls	OR (95% CI)	Negative FH Cases	Controls	OR (95% CI)
*Arg-A1*	0.607	0.711	Reference	0.695	0.705	Reference
*Arg-A2*	0.091	0.041	2.10 (1.08–4.06)	0.048	0.045	1.06 (0.53–2.12)
*Pro-A1*	0.150	0.111	1.49 (0.86–2.58)	0.098	0.119	0.80 (0.49–1.32)
*Pro-A2*	0.151	0.137	1.27 (0.72–2.24)	0.160	0.131	1.27 (0.83–1.95)

FH – family history; OR – odds ratio; CI – confidence interval

We examined the relationship between age at onset and genotypes and found a positive correlation in the FH group. The mean age of FH patients group with *A2A2 *genotype was 33.43 (± 8.08) years, whereas the mean age of patients with *A1A1 *and *A1A2 *genotypes was 42.44 (± 12.14) and 44.80 (± 10.85) years, respectively (Kruskal Wallis test p = 0.056). Therefore, the carrier's status of *A2A2 *genotype was associated with an earlier age at onset cancer with respect to the patients with *A1 *genotypes. However, this difference was in the frontier of statistically significant, possibly because of the smaller size of the group (7 patients to *Pro/Pro *genotype). No association was observed relating age at onset and *Arg72Pro *polymorphism (Kruskal Wallis test p = 0.747).

The analysis of the *TP53 *polymorphisms with respect to some clinical pathological showed a significant association of *Pro *or *A2 *genotypes with the presence of lymph node metastases (p = 0.009 and p < 0.001, respectively, adjusted for age and breast cancer family history, using logistic regression analysis).

## Discussion

Breast cancer is an heterogeneous disease, as sustained by wide variable morphological appearance, many risks factors and distinct gene expression profile [2,22]. Common genetic alterations (e.g. polymorphisms), with possible effects on function and/or protein expression, within genes involved in essential cellular pathways, such as carcinogen metabolism, DNA repair, cell cycle control and cell proliferation, could predispose individuals to cancer [15,23-25], including breast cancer [15,26-29].

The *TP53 *is one of the major tumour suppressor genes which carry out essential functions in preservation of genome integrity. Thus, when the cell is under stress, particularly stress which will involve DNA damage, p53 promotes growth arrest, allowing the cell to repair the DNA lesions. If the damage is excessively hazardous, then p53 will lead to cell apoptosis. Several genetic polymorphisms have been described in *TP53 *gene [18] and some of these variants seem to confer different functions among the p53 [30-32].

In the present study, we evaluated two separate *TP53 *polymorphisms, *Arg72Pro *and *PIN3 Ins16bp*, in two groups of breast cancer, familial and sporadic cases, as well as in matching control groups. The allelic frequencies of our control group for the different polymorphisms are in accordance with earlier reports from European populations [16,33]. We found a deviation from Hardy-Weinberg equilibrium in overall group of controls for *Arg72Pro *P53 polymorphism genotypes. Hardy-Weinberg equilibrium depends on a series of features about the tested population, including, for example the sample population size, random mating, no migration, no genetic drift and no selection taking place [34]. Thus, this deviation could be due to chance or violation of these assumptions, being the possibility of genotyping errors lower, since 10% our control sample genotyping were confirmed by direct sequence and its were similar with other European populations.

Concerning the *codon 72 TP53 *polymorphism (*Arg72Pro)*, we did not find any association between this polymorphism and breast cancer. Our results are in agreement with other studies [33,35,36], however, the literature remains highly controversial regarding the role of this polymorphism in breast cancer risk [37-42]. One study showed that *TP53 72Pro *variant induces transcription activation more efficiently than *TP53 72Arg *variant [40]. On the other hand, other authors revealed that *TP53 72Pro *variant induce cell cycle arrest better than *72Arg *[31]. Other studies have showed that *TP53 72Arg *variant is more efficient in inducing apoptosis [32,42]. Beside apoptosis and cell cycle control, p53 protein seems to be crucial in the regulation of the different DNA repair pathways [43]. A recent study demonstrated the influence of *TP53 Arg72Pro *in DNA repair capacity, showing that *TP53 72Pro *variant activates several *TP53 *dependent target genes involved in DNA repair and DNA damage repair much more efficiently than the *72Arg *variant expressing cells [30]. These contradictory results could be explained by the differential effects of this alteration in p53 function. Several *in vitro *evidences have demonstrated that both *TP53 Arg72Pro *variants may selectively regulate specific cellular functions.

In *TP53 PIN3 *polymorphism, our findings suggest an association of *A2A2 *genotype and increased breast cancer risk among women with FH and sporadic breast cancer, suggesting that this polymorphism contributed to enhance susceptibility for breast cancer among Portuguese population, regardless of the presence of FH. Our results are supported by previously reported studies suggesting an association of *PIN3 A2 *genotypes with breast cancer risk [44]. Although, the biological effect of the *TP53 PIN3 Ins16bp *polymorphism is currently unclear, theoretically, this polymorphism could affect mRNA splicing, altering the coding regions and therefore being implicated in regulation of gene expression and DNA-protein interactions, resulting in a defective protein [45,46]. Until now, just a single study had show *PIN3 A2 *allele presents reduced mRNA stability [47].

The linkage disequilibrium between *TP53 *polymorphisms region could be an important factor affecting the incidence of cancer in general [48,49], and breast cancer, in particular [42,44,50]. Thus, haplotype analysis would be important to confirm the significance of this variant on breast cancer susceptibility. A statistical significant association was found between *Arg-A2 *haplotype and breast cancer susceptibility among women with FH of breast cancer. On the other hand, a recent study has found that *Pro-A1 *haplotype individuals present increased breast cancer risk, however, in *BRCA2 *mutation carriers [42]. Nevertheless, other reports have also demonstrated a positive association of *Arg-A2 *haplotype with cancer [44,48]. Moreover, functional studies have shown that, in a specific haplotype combination, *A2 *allele is associated with decreased apoptotic and DNA repair capacity [33,48].

Our findings suggest the *Pro/Pro *and *A2A2 TP53 *genotypes as predictor factors for the presence of lymph node metastases, being in agreement with previously functional studies in the biological consequences of these variations in P53 protein functions [40,47].

The natural history of breast cancer can be influenced by several factors. We hypothesize that under the influence of *TP53 *genetic polymorphisms, chronic exposure to higher levels of several endogenous (e.g. estrogens) and exogenous breast carcinogens resulting in consequent higher accumulation of DNA damage during an individual's lifetime, may alter the age at onset of disease. Moreover, it has been suggested that *TP53 *polymorphisms are associated to familial breast cancer by the age of 50 years [33]. Our results are consistent with this hypothesis, since *TP53 PIN3 Ins16bp *polymorphism seems to influence directly the age to onset of familial breast cancer.

## Conclusion

In conclusion, our findings suggest *TP53 PIN3 Ins16bp *polymorphism as a real risk modifier in breast cancer disease, either in sporadic and familial breast cancer. Subsequently, these results will be crucial in the characterization of the genetic breast cancer susceptibility profile, within familial breast cancer cases non-carriers of *BRCA1/BRCA2 *mutations. Furthermore, our findings suggest *TP53 Arg72Pro *and *PIN3 Ins16bp *polymorphisms as predictive factors of presence of lymph node metastases.

## Abbreviations

FH: Family history; PCR: Polymerase chain reaction; RFLP: Restriction fragment length polymorphism; OR: Odds ratio; CI: Confidence interval; OS: Estimate overall survival.

## Competing interests

The author(s) declare that they have no competing interests.

## Authors' contributions

The SC was the principal investigator; contribute to data and samples collection, developed study design, experimental plan and implementation, statistical analysis, and drafted the manuscript. DP contributed to data and samples collection and critical revision of the manuscript. DP, HR and JCT were critical to data and samples collection. RM contributed to study design, samples collection, data analysis and critical review of the manuscript. FS contributed to conception of the study hypotheses, study design and critical review of the manuscript. In addition, all the authors read and approved the final submitted manuscript.

## Pre-publication history

The pre-publication history for this paper can be accessed here:


